# Accelerated Bone Regeneration by Astragaloside IV through Stimulating the Coupling of Osteogenesis and Angiogenesis

**DOI:** 10.7150/ijbs.57681

**Published:** 2021-04-24

**Authors:** Feng Wang, Huijuan Qian, Lingchi Kong, Wenbo Wang, Xiaoyu Wang, Ze Xu, Yimin Chai, Jia Xu, Qinglin Kang

**Affiliations:** Department of Orthopaedic Surgery, Shanghai Jiao Tong University Affiliated Sixth People's Hospital, Shanghai 200233, PR China.

**Keywords:** astragaloside IV, preosteoclast, angiogenesis, bone marrow mesenchymal stem cell, distraction osteogenesis

## Abstract

Both osteoblasts and preosteoclasts contribute to the coupling of osteogenesis and angiogenesis, regulating bone regeneration. Astragaloside IV (AS-IV), a glycoside of cycloartane-type triterpene derived from the Chinese herb *Astragalus membranaceus*, exhibits various biological activities, including stimulating angiogenesis and attenuating ischemic-hypoxic injury. However, the effects and underlying mechanisms of AS-IV in osteogenesis, osteoclastogenesis, and bone regeneration remain poorly understood. In the present study, we found that AS-IV treatment inhibited osteoclastogenesis, preserved preosteoclasts, and enhanced platelet-derived growth factor-BB (PDGF-BB)-induced angiogenesis. Additionally, AS-IV promoted cell viability, osteogenic differentiation, and angiogenic gene expression in bone marrow mesenchymal stem cells (BMSCs). The activation of AKT/GSK-3β/β-catenin signaling was found to contribute to the effects of AS-IV on osteoclastogenesis and osteogenesis. Furthermore, AS-IV accelerated bone regeneration during distraction osteogenesis (DO), as evidenced from the improved radiological and histological manifestations and biomechanical parameters, accompanied by enhanced angiogenesis within the distraction zone. In summary, AS-IV accelerates bone regeneration during DO, by enhancing osteogenesis and preosteoclast-induced angiogenesis simultaneously, partially through AKT/GSK-3β/β-catenin signaling. These findings reveal that AS-IV may serve as a potential bioactive molecule for promoting the coupling of osteogenesis and angiogenesis, and imply that AKT/GSK-3β/β-catenin signaling may be a promising therapeutic target for patients during DO treatment.

## Introduction

Distraction osteogenesis (DO) is a common trauma and orthopedic surgical procedure used for bone defect repair and osseous deformity correction 1-3, where gradual rhythmic traction is applied using an external fixator to fully induce neo-osteogenesis in the distraction zone between the proximal and distal bone segments 4, 5. Despite its unique osteogenesis-inducing ability, DO requires a lengthy consolidation phase with the heavy external fixator in position, which increases the patient's discomfort and complications 6. Therefore, accelerating mature callus formation and shortening the treatment duration are of great clinical significance.

Bone is a highly vascularized tissue, in which the abundant vessel networks not only provide access to the delivery of nutrients and oxygen, but also secrete specific cytokines, regulating a series of biological activities 7-10. Based on morphological, molecular, and functional criteria, Kusumbe et al. subdivided the capillaries in the skeletal system, into type-H and type-L endothelium 11. Type-H endothelial cells, characterized by high expression of platelet endothelial cell adhesion molecule-1 and endomucin (CD31^hi^EMCN^hi^), mediate local growth of the vasculature and provide niche signals for perivascular osteoprogenitors, enhancing the coupling of angiogenesis and osteogenesis 11, 12. Additionally, this specific capillary subtype is regulated by slit homolog 3 (slit3) secreted from osteoblasts, platelet-derived growth factor-BB (PDGF-BB) from preosteoclasts, and other cell-matrix signals in the microenvironment 13-16. Improved coupling of osteogenesis and angiogenesis, achieved by various interventions, is effective in bone defect and fracture healing, implant osseointegration, and osteoporosis prevention 14, 17-20, which highlights its therapeutic potential for accelerating bone regeneration in DO.

Astragaloside IV (AS-IV) is a glycoside of cycloartane-type triterpene and the main active component in the medicinal plant *Astragalus membranaceus*, which has been widely used in traditional Chinese medicine to treat wounds, anemia, and chronic fatigue 21, 22. AS-IV has diverse biological activities, including promoting angiogenesis, ameliorating ischemia/reperfusion injury, and regulating glucose and lipid metabolism 23-25. Additionally, AS-IV has been reported to improve behavioral and neuro-chemical deficits largely due to its antioxidant, antiapoptotic, and anti-inflammatory properties 26. Meanwhile, AS-IV has been reported to inhibit osteoclastogenesis 27, and to promote the osteogenic differentiation of OCT-1 cells by combining with centrifugation pressure 28. Although the results of above studies imply the potential regulatory effect of AS-IV on the coupling of osteogenesis and angiogenesis, the underlying mechanisms involved are unexplored, and the biological functions of AS-IV in bone regeneration remain to be investigated. Of note, most biological activities of AS-IV are conducted through the activation of protein kinase B (AKT) 23-25. AKT, in turn, activates Wingless/Integrated (Wnt) pathway through the activation and nuclear translocation of β-catenin, which inhibits osteoclastogenesis and promotes osteogenesis 29-31, by phosphorylating Ser9 and inactivating glycogen synthase kinase-3β (GSK-3β) 32. Based on this, AS-IV was hypothesized to promote preosteoclast PDGF-BB-induced angiogenesis by inhibiting the formation of multinucleated mature osteoclasts, and stimulate the osteogenic differentiation of bone marrow-derived mesenchymal stem cells (BMSCs) simultaneously. Together with its direct stimulating effect on angiogenesis 23, AS-IV may have enormous potential to serve as an effective therapeutic agent for accelerating bone regeneration in DO.

The present study investigated the ability of AS-IV to increase PDGF-BB-stimulated angiogenesis by preserving the preosteoclasts. In addition, the *in vitro* effects of AS-IV on the viability, osteogenic differentiation, and angiogenic gene expression of BMSCs were explored. Furthermore, role of the AKT/GSK-3β/β-catenin pathway in regulating the effects of AS-IV on osteoclastogenesis and osteogenesis was investigated. Finally, a rat DO model was employed to examine the* in vivo* effects of AS-IV on bone regeneration and the coupling of osteogenesis and angiogenesis.

## Methods

### Cell Culture

BMSCs were freshly harvested from the bone marrow of 2-week-old Sprague-Dawley (SD) male rats by flushing the bone marrow from femurs and tibias. Flushed bone marrow cells were cultured in modified Eagle's medium alpha (HyClone, USA) supplemented with 10% (v/v) fetal bovine serum (FBS; Gibco, USA) and 1% (v/v) penicillin-streptomycin (P/S; Gibco). At 80%-90% confluence, the cells were treated with trypsin (Gibco) and re-plated for expansion. The BMSCs between passages 2 and 5 were used in the downstream experiments. Cells from the macrophage cell line, RAW264.7 and endothelial cell line, Ea.hy926 were cultured in high glucose Dulbecco's Modified Eagle's Medium (HyClone) containing 10% (v/v) FBS and 1% (v/v) P/S. All cells were cultured at 37 °C in a humidified atmosphere with 5% CO_2_.

### Preparation of the conditioned medium (CM)

AS-IV was dissolved in dimethyl sulfoxide (DMSO; Sigma-Aldrich, USA) at a final concentration below 0.1% (v/v). The RAW264.7 cells were inoculated into 24-well plates (1.5×10^4^/well) and treated with 100 ng/mL receptor activator for nuclear factor κB ligand (RANKL, PeproTech, USA) and serial concentrations of AS-IV (Sigma-Aldrich). The medium was replenished every 2 days. After 4 days of induction, the medium was replaced with a complete medium for a subsequent 2 days of incubation. Then, the CM from each group was harvested and centrifuged at 2000 ×g for 10 min to collect the supernatant, which was stored at -80 °C for further experiments.

### Tartrate-resistant acid phosphatase (TRAP) staining

After 4 days of induction, RAW264.7 cells were fixed with 4% (w/v) paraformaldehyde (PFA; Servicebio, China) for 15 min, washed three times with phosphate-buffered saline (PBS; HyClone), and then stained for TRAP according to the manufacturer's instructions (387A, Sigma-Aldrich). TRAP^+^ mononuclear cells and multinucleated cells containing at least three nuclei were identified as preosteoclasts and osteoclasts, respectively. The number of preosteoclasts was counted and the osteoclast area was measured using ImageJ software (National Institutes of Health, USA).

### Enzyme-linked immunosorbent assay (ELISA)

PDGF-BB concentration analyses of the CM were performed using a mouse PDGF-BB ELISA kit (Proteintech, USA). All the procedures were conducted according to the manufacturer's instructions, and the protein concentration was calculated according to a standard curve.

### Angiogenesis-related assays *in vitro*

The cells of the endothelial cell line Ea.hy926 were cultured under different treatment conditions: 1) CM^Control^ group (CM from uninduced RAW264.7 cells and isotype IgG); 2) CM^RANKL^ group (CM from RANKL-induced RAW264.7 cells and isotype IgG); 3) CM^RANKL+AS-IV^ group (CM from RANKL + AS-IV-treated RAW264.7 cells and isotype IgG); and 4) CM^RANKL+AS-IV^ + PDGF-BB neutralizing antibody group. PDGF-BB-neutralizing antibody and IgG isotype control antibody were purchased from the R&D Systems (USA).

For the tube formation assay, Ea.hy926 cells (2×10^4^/well) were seeded onto 96-well plates coated with Matrigel (BD Biosciences, USA) and incubated in a blank medium under different treatment conditions for 6 h. The total tube length and total branch points were measured to evaluate the tube formation ability.

For the transwell migration assay, Ea.hy926 cells (2×10^4^/well) were loaded into the top chamber of a 24-well, 8μm pore-size transwell plate (Corning, USA), and then incubated with CM from different groups with PDGF-BB-neutralizing antibody or isotype IgG in the lower chamber for 4 h. Subsequently, unmigrated cells that remained in the upper chambers were removed with cotton swabs, while the migrated cells that passed through the membrane pores were fixed with 4% (w/v) PFA for 15 min and stained with 0.1% (w/v) crystal violet (Solarbio, China) for 5 min. The number of migrated cells was counted using the ImageJ software.

For the scratch wound assay, Ea.hy926 cells were seeded into 6-well plates and cultured until confluence. Next, the confluent layers of cells were scratched using a sterile pipette tip. After washing, the cells were incubated in a blank medium under different treatment conditions. Images of the wounds were acquired immediately, 6 h, and 12 h later, the wound areas were measured using the ImageJ software, and the migration area (%) was calculated.

### Cell Viability Assay

The viability of BMSCs was determined using the CCK-8 assay. Briefly, BMSCs were inoculated in 96-well plates (5000 cells/well) and incubated in the presence of serial concentrations of AS-IV. After incubation for 24, 48, or 72 h, 90 μL of fresh culture medium and 10 μL CCK-8 reagent (Dojindo, Japan) were added into each well and incubated for another 2 h. Then, the optical density (OD) was measured at 450 nm using a microplate reader (Thermo, USA). Additionally, MK2206 (100 nM; MedChemExpress, USA) was administered to verify the molecular mechanism by which AS-IV regulates cell viability of BMSC.

### Osteogenic Differentiation

To determine the effects of AS-IV on the osteogenic differentiation of BMSCs, alkaline phosphatase (ALP) and mineral deposition were detected. Briefly, the BMSCs were inoculated in 24-well plates (5×10^4^/well). Upon 80% confluence, the medium was replaced with osteogenic induction media (OIM, 20 mM β-glycerophosphate, 1 nM dexamethasone, and 50 μM L-ascorbic acid-2-phosphate in complete medium; Sigma-Aldrich) containing serial concentrations of AS-IV, and the medium was replenished every 2 days. In another series of experiments, MK2206 (100 nM, MedChemExpress) was administered to verify the molecular mechanism by which AS-IV regulates osteogenic differentiation of BMSCs. ALP staining and activity assays were performed 7 days after osteogenic induction according to the manufacturer's instructions (Beyotime, China). On the 14th day of differentiation, alizarin red S staining (ARS; Cyagen Biosciences, China) was performed to evaluate mineral deposition. For quantitative analysis of the mineralization, the deposited calcium was eluted with 10% (w/v) cetylpyridinium chloride (Sigma-Aldrich), and the OD value was measured at 570 nm.

### Quantitative real-time polymerase chain reaction (qRT-PCR) analysis

Total RNA was extracted using an RNA Purification Kit (EZBioscience, USA) and cDNA was obtained from 500 ng of total RNA using the Reverse Transcription Kit (EZBioscience). Next, qRT-PCR was performed using SYBR Green qPCR Master Mix (EZBioscience). Relative gene expression was calculated using the 2^-ΔΔCT^ method, and *GAPDH* was used as a reference for normalization. The primers were purchased from BioTNT, and primer sequences are shown in Table 1.

### Western blot analysis

Total protein was extracted using the RIPA lysis buffer with protease inhibitor and protein phosphatase inhibitor (Solarbio) at 4 °C. Protein concentration was determined using a BCA Protein Assay Kit (EpiZyme, China). Equal amounts of protein (30 μg) were subjected to 10% (w/v) SDS-PAGE and then transferred to a polyvinylidene difluoride membrane (Millipore, USA). After blocking with 5% (w/v) bovine serum albumin (BSA), the membrane was incubated with primary antibodies at 4 °C overnight. Afterward, the membrane was incubated with horseradish peroxidase (HRP)-conjugated secondary antibodies (1:10000; 111-035-003, Jackson ImmunoResearch, USA) at room temperature for 1 h. Immunoreactive bands were visualized using enhanced chemiluminescence reagent (Millipore) and the grayscale of protein bands were semi-quantified using the ImageJ software.

Primary antibodies used in this study included anti-AKT (1:1500; #4691, Cell Signaling Technology, USA), anti-phosphorylated AKT (p-AKT, Ser473; 1:1000; #4060, Cell Signaling Technology), anti-GSK-3β (1:1500; #12456, Cell Signaling Technology), anti-phosphorylated GSK-3β (p-GSK-3β, Ser9; 1:1000; #5558, Cell Signaling Technology), anti-β-catenin (1:1500; #8480, Cell Signaling Technology), anti-phosphorylated β-catenin (p-β-catenin, Ser675; 1:1000; #4176, Cell Signaling Technology), anti-NFATc1 (1:1000; A1539, ABclonal Technology, China), and anti-GAPDH (1:2000; #5174, Cell Signaling Technology).

### Immunofluorescence staining

BMSCs were fixed with 4% (w/v) PFA and permeabilized with 0.1% (v/v) Triton X-100 in PBS containing 5% (w/v) BSA. After blocking with 5% (w/v) BSA for 1 h, the cells were incubated with anti-β-catenin antibody (1:100; #8480, Cell Signaling Technology) overnight at 4 °C. Subsequently, the cells were washed thrice with PBS and incubated with a FITC-conjugated secondary antibody (1:1000; ab6717, Abcam, UK) for 1 h, followed by incubation with 4',6-diamidino-2-phenylindole (DAPI) for 15 min. The fluorescence signal was captured using a fluorescence microscope (DMi8, Leica, Germany).

### Animal surgery and treatment

All experimental procedures were approved by the Animal Welfare Ethics Committee of Shanghai Jiao Tong University Affiliated Sixth People's Hospital (DWLL2021-0413). A total of 36 adult male SD rats (350-400 g) were used in this study and randomly assigned to the control (n = 18) and AS-IV (n = 18) groups. To establish the DO model, a transverse osteotomy was performed at the midshaft of the right tibia after anesthesia and exposure. Then, a specially designed monoliteral external fixator (Xinzhong Company, China) was mounted to fix the proximal and distal segments of the tibia. Thereafter, surgical incisions were closed layer-wise. The periosteum was preserved as much as possible during the procedure. The DO procedures comprised three phases: latency phase for 5 days, distraction phase for 10 days (0.25 mm every 12 h), and the consolidation phase for 4 weeks. AS-IV (20 mg/kg/day) and equal-volume vehicle (0.5% carboxymethyl cellulose solution; w/v) were intragastrically administered during the consolidation phase.

### Digital radiography and micro-computed tomography (CT)

From the beginning of the consolidation phase, X-ray films focusing on the distraction gap were acquired weekly. The lengthened tibia specimens were harvested 2 (n = 6) and 4 (n = 6) weeks after distraction. Micro-CT (Skyscan 1172, Bruker, Germany) with a voltage of 80 kV, a current of 112 μA, and an exposure time of 370 ms was utilized to quantitatively evaluate bone regeneration in the distraction zone. Thereafter, three-dimensional (3D) reconstructions of the regenerated callus were produced using the CTVox software (Bruker). Parameters, including bone volume/tissue volume (BV/TV) and bone mineral density (BMD), of the regenerated bone were analyzed using the CTAn software (Bruker).

### Microfil perfusion

To evaluate the vascularization of the distraction area, animals (n = 3) were randomly selected and subjected to Microfil perfusion 2 weeks after distraction. Briefly, after general anesthesia, the thorax was exposed. Then, the descending aorta was ligated, and the aorta was intubated with a catheter from the left ventricle, followed by incision of the right auricle. Heparinized saline and Microfil (Flowtech, USA) were perfused from the left ventricle to the circulatory system. Subsequently, the rats were placed at 4 °C for 24 h to ensure complete gelatinization of Microfil. The tibia samples were fixed in 4% (w/v) PFA for 24 h, decalcified in 10% (w/v) ethylenediamine tetraacetic acid (EDTA, pH 7.4) for 21 days, and employed for micro-CT analyses with an isotropic resolution of 9 μm, followed by three-dimensional reconstruction and analyses of blood vessel volume using the CTVox and CTAn software packages.

### Biomechanical testing

The mechanical characteristics of the fresh tibia specimens (n = 6) were determined using a four-point bending device (Hounsfield Test Equipment, UK), 4 weeks after consolidation. During the test, the tibia specimens were loaded in the anterior-posterior direction with the posterior side in tension. The modulus of elasticity (E-modulus), ultimate load, and energy to failure were analyzed using Vernier Graphical Analysis software (Vernier, USA).

### Histological, immunohistochemical and immunofluorescent analyses

For histological analyses, after 2 (n = 3) and 4 (n = 3) weeks of consolidation, the tibia specimens were fixed in 4% (w/v) PFA for 24 h, decalcified in 10% (w/v) EDTA for 21 days, dehydrated through graded ethanol of increasing concentration, and then embedded in paraffin. Samples were cut into 5-μm-thick longitudinally oriented sections and processed for hematoxylin-eosin (H&E), Masson's trichrome, and Safranine O-Fast Green (SO-FG) staining.

For immunohistochemical staining, sections were incubated in 0.3% (v/v) hydrogen peroxide for 20 min to quench endogenous peroxidase activity. After antigen retrieval in 0.01 mol/L citrate buffer (pH 6.0) at 65 °C for 20 min and blocking with 5% (v/v) goat serum for 1 h, sections were incubated with primary antibodies at 4 °C overnight. After incubation with secondary antibodies (1:1000; 111-035-003, Jackson ImmunoResearch) conjugated with HRP at room temperature for 1 h, an HRP-streptavidin system (Dako, Denmark) was used to detect positive areas followed by counterstaining with hematoxylin. Primary antibodies used in this study included anti-OCN (1:100; A6205, ABclonal, China) and anti-β-catenin (1:100; #8480, Cell Signaling Technology).

### Immunofluorescent analysis

CD31 and EMCN double immunofluorescence staining were performed to evaluate the extent of type-H vessel formation. After 2 weeks of consolidation, tibia specimens (n = 3) were decalcified in 18% (w/v) EDTA (pH 7.4) for 7 days after fixation. Subsequently, the samples were dehydrated in 30% (w/v) sucrose, embedded in optimal cutting temperature compound, and cut into 10 μm thick longitudinally oriented sections. After blocking with 5% (w/v) BSA for 1 h, bone sections were incubated with primary antibodies overnight at 4 °C, followed by incubation with fluorophore-conjugated secondary antibodies (1:200; ab97035, ab6840, Abcam) at room temperature for 1 h. Nuclei were stained with DAPI. A fluorescence microscope was used for observation and image capture. The EMCN antibody was purchased from Santa Cruz Biotechnology (1:100; sc-65495, USA). The CD31 antibody and secondary antibodies were obtained from Abcam (1:100; ab64543).

### Statistical analysis

All data are presented as mean ± standard deviation. The statistical differences were analyzed with Student's *t*-test between two groups or one-way analysis of variance (ANOVA) followed by Tukey's *post hoc* test among groups using the GraphPad Prism 8 software (GraphPad Software, USA). A two-tailed *P*-value of less than 0.05 was considered statistically significant.

## Results

### AS-IV preserved preosteoclasts and the production of PDGF-BB through the AKT/GSK-3β/β-catenin pathway

**Figure 1:**
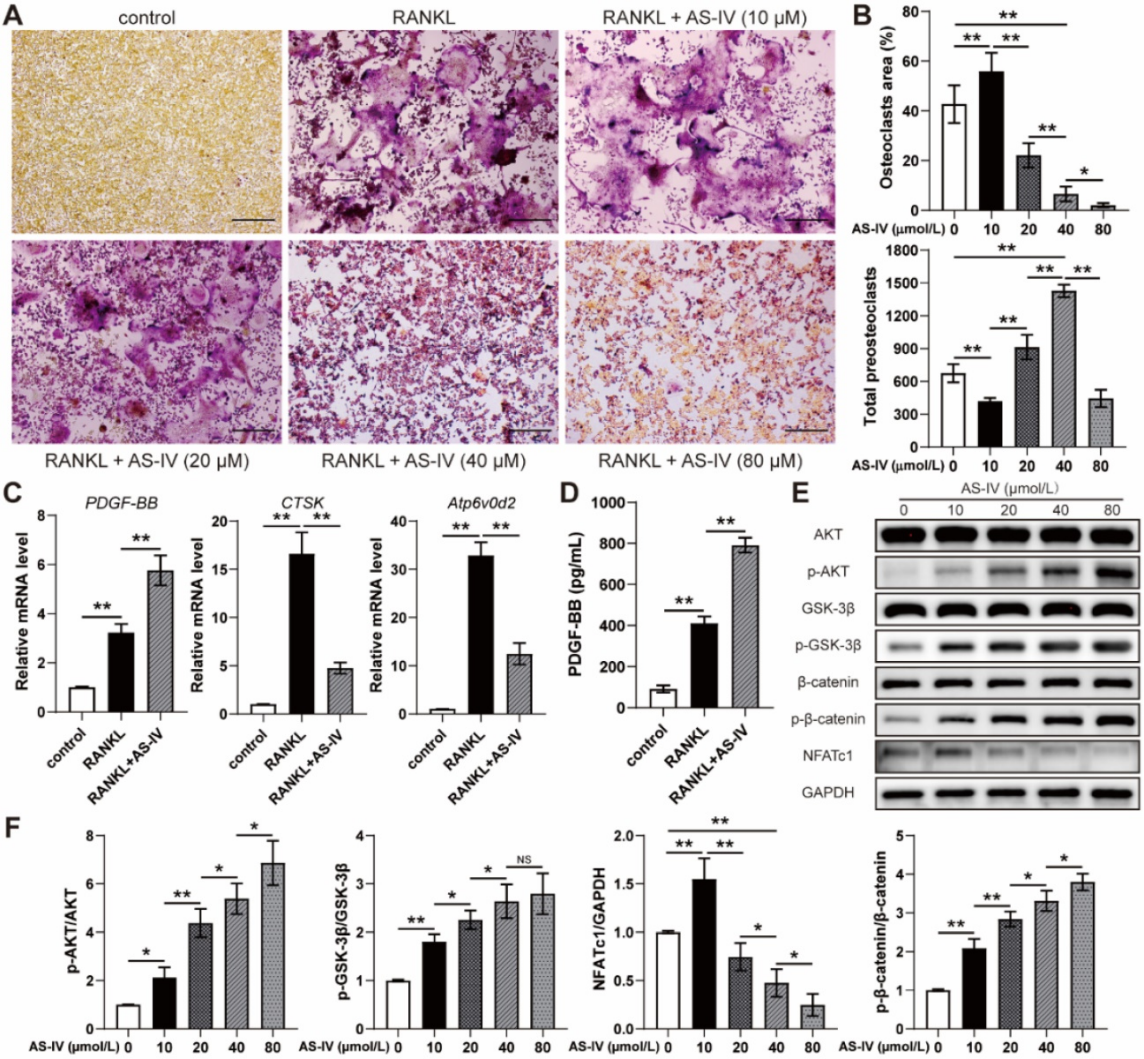
** Effect of AS-IV on osteoclast differentiation of RAW264.7 cells induced by RANKL. (A)** Representative images of TRAP staining showing osteoclast and preosteoclast formation from RAW264.7 cells treated with vehicle, RANKL, and RANKL + various concentrations of AS-IV. Scale bar: 200 µm. **(B)** Quantitative analysis of the area of osteoclasts and the number of preosteoclasts. **(C)** qRT-PCR analysis of *CTSK*, *Atp6v0d2*, and *PDGF-BB* expression levels in RAW264.7 cells treated with vehicle, RANKL, and RANKL + AS-IV (40 µM). **(D)** Detection of PGDF-BB concentration in conditioned media from RAW264.7 cells treated with vehicle, RANKL and RANKL + AS-IV (40 µM) by ELISA. **(E)** Western blot of AKT, p-AKT, GSK-3β, p-GSK-3β, β-catenin, p-β-catenin, and NFATc1 in RAW264.7 cells treated with vehicle, RANKL, and RANKL + various concentrations of AS-IV. **(F)** Quantitative analysis of the phosphorylated levels of AKT, GSK-3β, and β-catenin and the protein level of NFATc1 relative to GAPDH. The data were confirmed by one-way analysis of variance (ANOVA) followed by Tukey's *post hoc* test and are presented as the means ± SD. *P < 0.05; ^**^P < 0.01.

To examine the effects of AS-IV on osteoclastogenesis, the cells of the osteoclast precursor cell line RAW264.7 were induced by RANKL and treated with serial concentrations of AS-IV for 4 days. TRAP staining showed that AS-IV promoted osteoclast differentiation at low concentrations (10 μM); on the contrary, osteoclastogenesis was inhibited at higher concentrations with a reduction in the osteoclast area and accumulation of preosteoclasts (Figure 1A, B). However, high concentrations (80 μM) of AS-IV excessively inhibited osteoclastogenesis, reducing the area of osteoclasts and the number of preosteoclasts synchronously (Figure 1A, B). Hence, the expression levels of genes related to osteoclastogenesis of RAW264.7 cells in the 40 μM group were assessed with qRT-PCR analysis. The results showed that RANKL-induction significantly increased *Atp6v0d2* and *CTSK* mRNA levels compared to the levels observed in the uninduced group, while the effect was markedly repressed by AS-IV treatment (Figure 1C). However, *PDGF-BB* gene expression was elevated in the RANKL+AS-IV group (Figure 1C). Moreover, the results of the ELISA showed that the production of PDGF-BB from RANKL-induced macrophages was much higher than that in the uninduced group, and the AS-IV treatment further increased the concentration of PDGF-BB in the CM (Figure 1D). NFATc1 and β-catenin are both downstream to AKT signaling. Based on the western blot results, AS-IV activated AKT, which was evident through the elevated level of AKT phosphorylation, in the RANKL-induced RAW264.7 cells (Figure 1E, F). The expression of NFATc1 increased at low concentrations of AS-IV but decreased at higher concentrations (Figure 1E, F). However, AS-IV activated β-catenin in the macrophages in a dose-dependent manner through the AKT/GSK-3β pathway (Figure 1E, F). Therefore, our results suggested that AS-IV increased the production of PDGF-BB by preserving preosteoclasts under RANKL induction, and this effect was attributed to the activation of the AKT/GSK-3β/β-catenin pathway.

### AS-IV promoted preosteoclast PDGF-BB-induced angiogenesis

**Figure 2:**
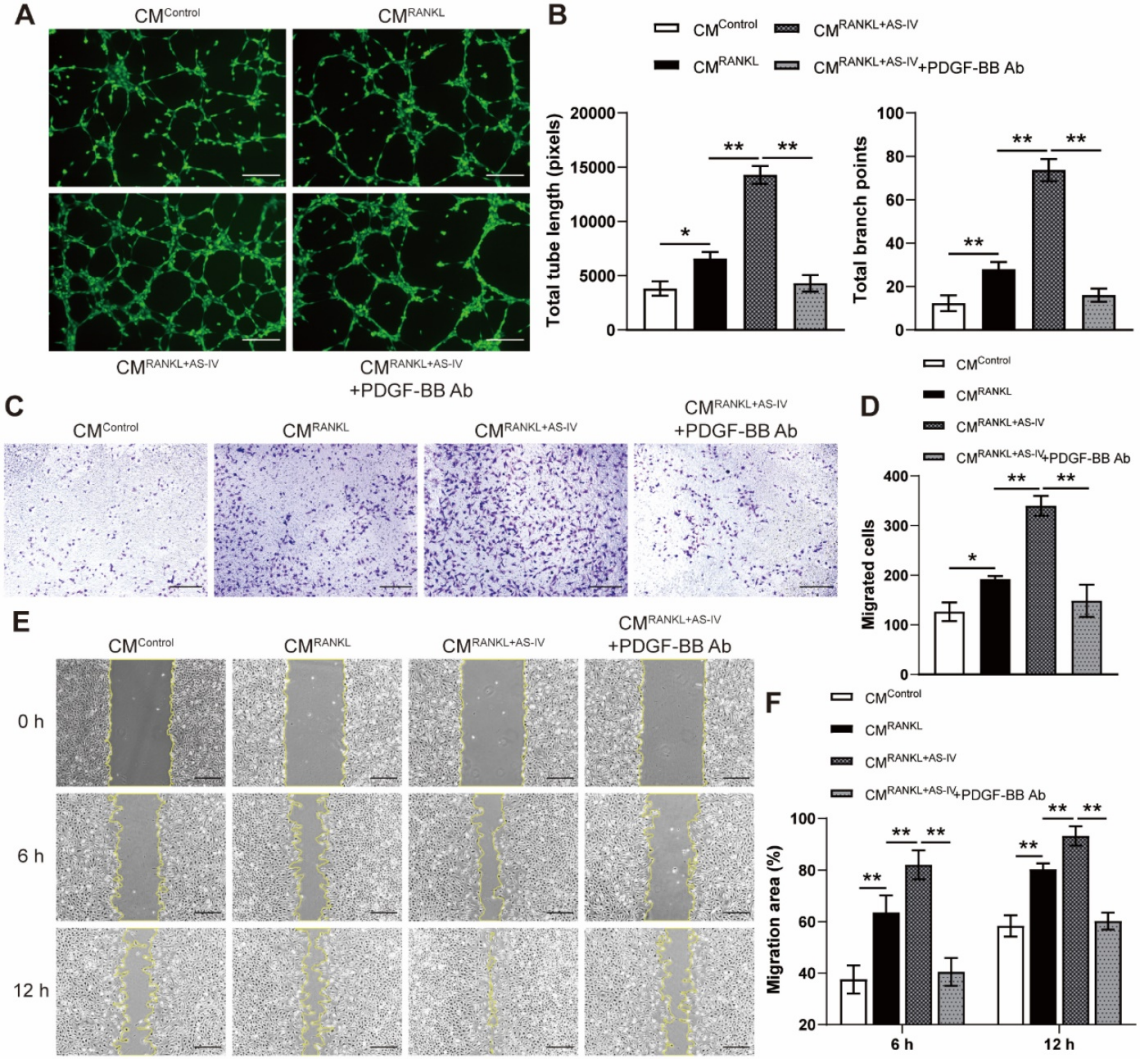
** AS-IV augments the pro-angiogenic effects of preosteoclasts on endothelial cells. (A, B)** Representative images (A) and quantification of tube formation (B) in Ea.hy926 cells stimulated with CM from each group and PDGF-BB-neutralizing antibody of IgG isotype control antibody. Scale bar: 200 µm. **(C-F)** Endothelial cell motility in different treatment groups was evaluated using the Transwell migration assay (C, D) and the scratch wound assay (E, F). Scale bar: 200 µm. The data were confirmed by one-way analysis of variance (ANOVA) followed by Tukey's *post hoc* test and are presented as the means ± SD. *P < 0.05; ^**^P < 0.01.

To elucidate the effects of AS-IV on preosteoclast-induced angiogenesis, Ea.hy926 cells were incubated with CM from each group for a series of angiogenesis-related functional assays. Endothelial cells treated with CM^RANKL^ formed a higher number of capillary tube-like structures, with increased total tube length and branch points, compared with those treated with CM^Control^, and AS-IV treatment further enhanced the angiogenic effects of CM from RANKL-induced macrophages (Figure 2A, B). However, PDGF-BB neutralizing antibodies markedly reduced the stimulating effect of CM^RANKL+AS-IV^ on tube formation (Figure 2A, B). The results of the transwell assay and scratch wound assay revealed that the CM from RANKL + AS-IV-treated macrophages induced the most optimal endothelial cell migration (Figure 2C-F). These results indicated that CM^RANKL+AS-IV^-induced promotion of angiogenesis was mostly attributable to the elevated production of preosteoclast PDGF-BB by AS-IV treatment.

### AS-IV enhanced cell viability, osteogenic differentiation, and angiogenic gene expression of BMSCs

**Figure 3:**
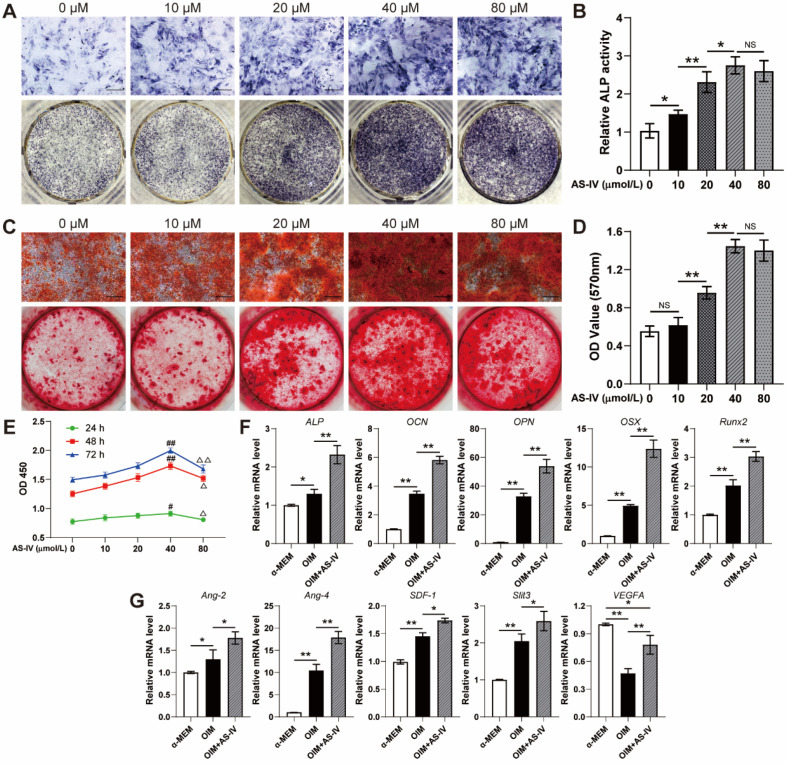
**AS-IV promotes osteogenic differentiation, cell viability, and the expression of angiogenic genes of BMSCs. (A-D)** Osteogenesis of BMSCs treated with OIM and different concentrations of AS-IV were determined with ALP staining (A), ALP activity assays (B) and alizarin red staining (C). Calcium deposition was assessed by measuring the optical density (D). Scale bar: 200 µm. **(E)** CCK-8 analysis of BMSC proliferation in different treatment groups. **(F, G)** Expression of osteogenic-specific genes (F) and angiogenic-specific genes (G) of BMSCs treated with OIM+AS-IV (40 µM) were assessed with qRT-PCR. The data were confirmed by one-way analysis of variance (ANOVA) followed by Tukey's *post hoc* test and are presented as the means ± SD. *P < 0.05; ^**^P < 0.01; ^#^*P* < 0.05 vs 0 µmol/L group; ^##^*P* < 0.01 vs 0 µmol/L group; ^Δ^*P* < 0.05 vs 40 µmol/L group; ^ΔΔ^*P* < 0.01 vs 40 µmol/L group.

To evaluate the effect of AS-IV on the osteogenic differentiation of BMSCs, the staining, and activity assay of ALP, an early osteogenic differentiation marker, was performed on the 7^th^ day of osteogenic induction. AS-IV (0-40 μM) significantly enhanced the staining and activity of ALP in a dose-dependent manner, whereas BMSCs treated with a higher concentration of AS-IV (80 μM) did not exhibit further enhanced ALP staining and activity compared to those in the 40 μM group (Figure 3A, B). The results of ARS staining and calcium quantitative analysis subsequently confirmed that AS-IV at a concentration of 40 μM induced maximal mineralization (Figure 3C, D). As evident from the CCK-8 assay, the BMSCs showed optimal cell viability when treated with AS-IV at 40 μM, while a further increase in concentration attenuated this effect (Figure 3E). Consequently, the optimal concentration of AS-IV was determined at 40 μM in the present study for further examination on the expression of osteogenic genes. As revealed by qRT-PCR, the expression levels of *ALP*, *OSX*, *OCN*, *OPN*, and *Runx2* in OIM-induced BMSCs were significantly increased by AS-IV treatment (Figure 3F). Moreover, the gene expression of several angiogenic factors in BMSCs was examined after osteogenic induction. Higher gene expression of *Ang-2*, *Ang-4*, *SDF-1*, and* Slit3*, a novel angiogenic factor related to type H vessels, was observed in the OIM group, and the expression of these genes was further elevated by AS-IV (40 μM) treatment (Figure 3G). However, the gene expression of *VEGFA*, another angiogenic factor associated with type-H endothelial phenotype, decreased after osteogenic induction (Figure 3G). Interestingly, the AS-IV treatment partially rescued *VEGFA* gene expression even though the osteogenic differentiation of BMSCs was simultaneously enhanced by AS-IV treatment (Figure 3G).

### AS-IV activated the AKT/GSK3β/β-catenin pathway in BMSCs

**Figure 4:**
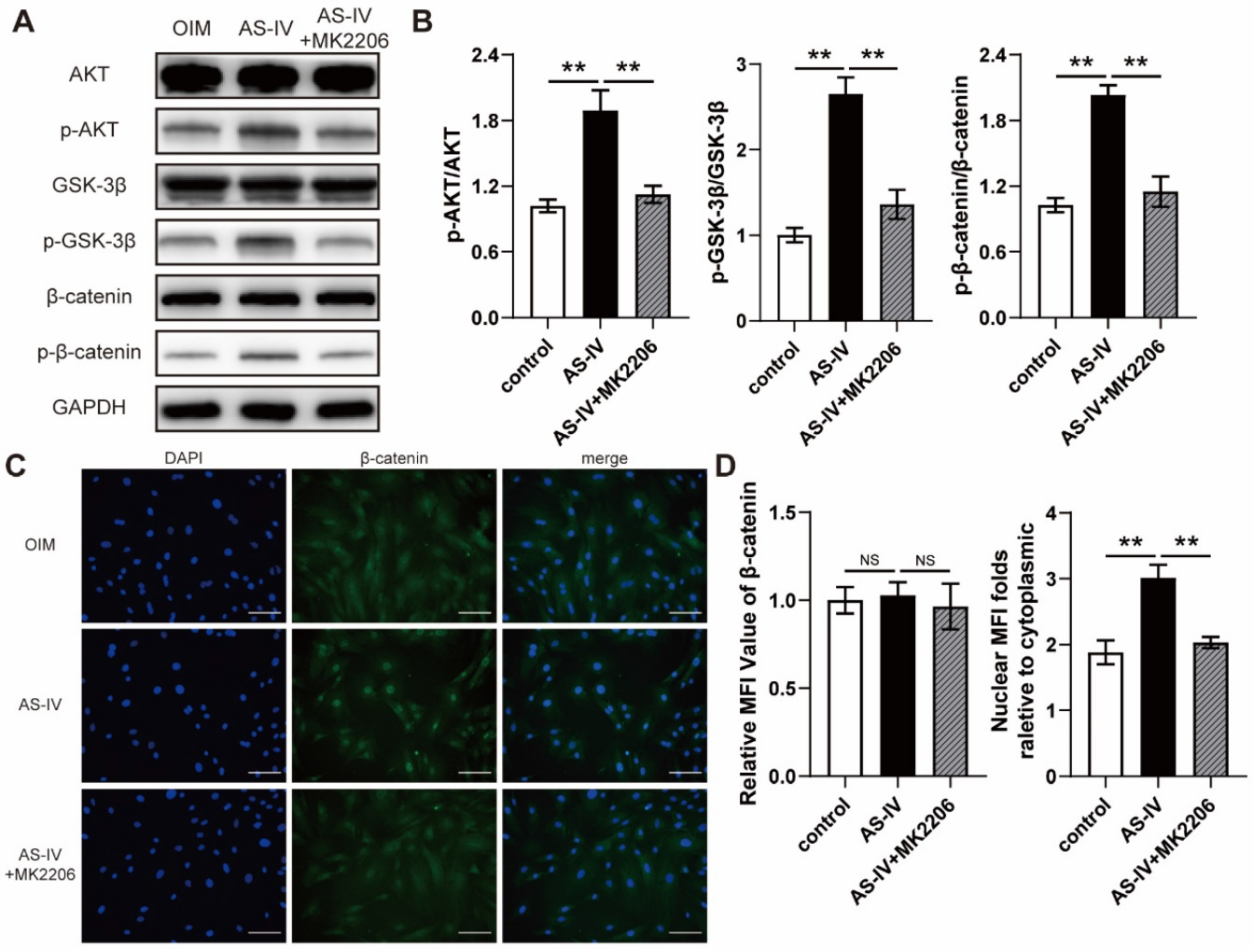
** AS-IV activates the AKT/GSK-3β/β-catenin pathway in BMSCs. (A)** Western blot of AKT, p-AKT, GSK-3β, p-GSK-3β, β-catenin, and p-β-catenin in BMSCs treated with OIM, OIM+AS-IV, and OIM+AS-IV+MK2206. **(B)** Quantitative analysis of the phosphorylated levels of AKT, GSK-3β, and β-catenin. **(C)** Representative immunocytochemistry images showing the expression and nuclear translocation of β-catenin in BMSCs treated with OIM, OIM+AS-IV, and OIM+AS-IV+MK2206. Scale bar: 100 µm. **(D)** Quantitative analysis of the expression and nuclear translocation of β-catenin. ^**^P < 0.01. MFI, mean fluorescence intensity. The data were confirmed by one-way analysis of variance (ANOVA) followed by Tukey's *post hoc* test and are presented as the means ± SD. ^NS^*P* > 0.05; ^**^P < 0.01.

**Figure 5:**
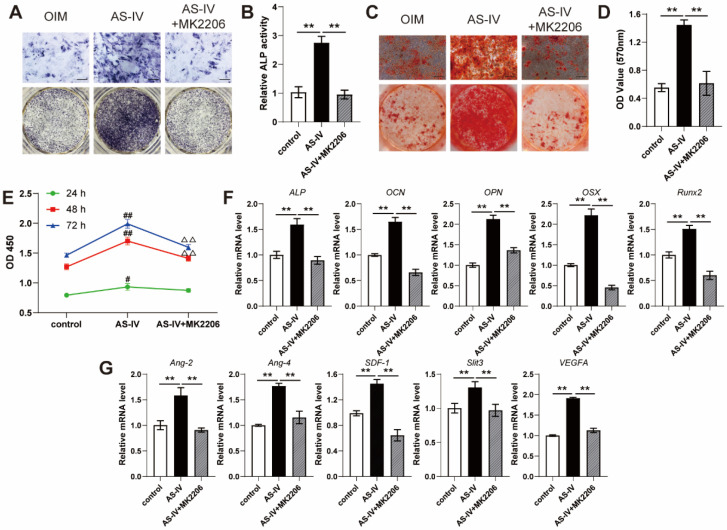
** MK2206 inhibits the effects of AS-IV on osteogenic differentiation, cell viability, and angiogenic gene expression of BMSCs. (A-D)** Osteogenesis of BMSCs treated with OIM, OIM+AS-IV, and OIM+AS-IV+MK2206 was determined with ALP staining (A), ALP activity assays (B) and alizarin red staining (C). Calcium deposition was assessed by measuring the optical density (D). Scale bar: 200 µm. **(E)** CCK-8 analysis of BMSC proliferation in different treatment groups. **(F, G)** Expression of osteogenic-specific genes (F) and angiogenic-specific genes (G) of BMSCs in different treatment groups were assessed using qRT-PCR. The data were confirmed by one-way analysis of variance (ANOVA) followed by Tukey's *post hoc* test and are presented as the means ± SD. ^**^P < 0.01; ^#^*P* < 0.05 vs control group; ^##^*P* < 0.01 vs control group; ^ΔΔ^*P* < 0.01 vs AS-IV group.

AKT is regarded as a vital upstream signal of Wnt/β-catenin, a classical pathway regulating osteogenic differentiation, due to its ability to prevent GSK-3β-mediated degradation of β-catenin. The results of the western blotting suggested that AS-IV (40 μM) activated AKT signaling in OIM-induced BMSCs, similar to the pharmacological mechanism in RANKL-induced RAW264.7 cells, and upregulated the phosphorylation levels of GSK3β and β-catenin (Figure 4A, B). However, MK2206, a specific inhibitor of AKT, significantly attenuated the AS-IV-induced activation of the AKT/GSK-3β/β-catenin pathway (Figure 4A, B). This significant variation in the activation state of β-catenin induced by AS-IV and MK2206 was further confirmed by immunofluorescence analysis. The results showed an elevated nuclear mean fluorescence intensity (MFI) relative to cytoplasm, which presented an optimal activation and nuclear translocation of β-catenin, in the AS-IV group and attenuated MFI folds in the AS-IV + MK2206 group (Figure 4C, D). Furthermore, MK2206 administration markedly attenuated the stimulatory effect of AS-IV on cell viability and osteogenic differentiation of BMSCs, as revealed by impaired ALP activity, mineralization, CCK-8 parameters, and osteogenic gene expression (Figure 5A-F). In addition, the AS-IV-improved angiogenic gene expression was also attenuated by MK2206 administration (Figure 5G). Taken together, these findings indicate that AS-IV enhanced cell viability, osteogenic differentiation, and angiogenic gene expression of BMSCs by stimulating the AKT/GSK-3β/β-catenin pathway.

### AS-IV accelerated bone consolidation during distraction osteogenesis

**Figure 6:**
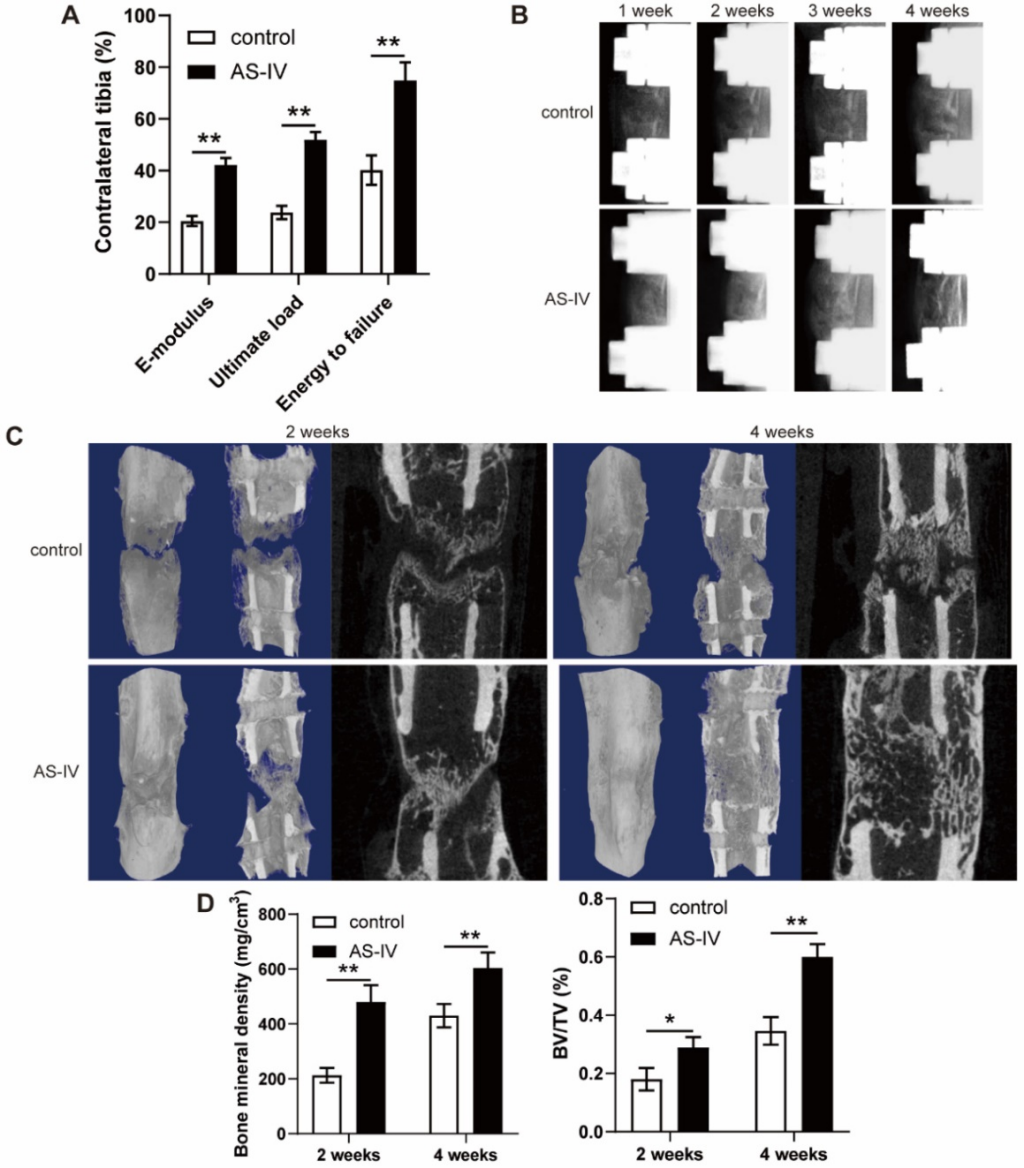
** AS-IV administration accelerates bone consolidation during distraction osteogenesis in rats. (A)** Mechanical properties, including E-modulus, ultimate load, and energy to failure of distraction regenerates in control and AS-IV groups. The values were normalized to the corresponding contralateral normal tibias. **(B)** Representative X-rays of distraction regenerates at various time points. **(C, D)** Representative 3D and longitudinal images (C) and quantitative analysis (D) of micro-CT data, including bone mineral density and bone volume/tissue volume, of the tibial distraction zone after 2 and 4 weeks of consolidation and treatment. The data were confirmed by Student's *t*-test between control group and AS-IV group and are presented as the means ± SD. ^*^P < 0.05; ^**^P < 0.01.

The fresh tibia specimens exhibited improved ultimate load, energy to failure, and elasticity modulus in the AS-IV group (Figure 6A). Additionally, a representative series of X-ray films across the time course of DO showed progression of bone consolidation (Figure 6B). At the beginning of the consolidation phase, little callus formation was observed in the distraction gap in both groups (Figure 6B). Over time, bone formation increased in both groups, while more opaque calli appeared in the AS-IV group compared to the control group in terms of volume and continuity of the callus, especially at the end of the consolidation phase (Figure 6B). Similar observations were confirmed by micro-CT examination of distraction regenerates at 2 and 4 weeks after distraction (Figure 6C). The BMD and BV/TV of distraction regenerates in the AS-IV group were significantly higher than those in the control group after 2 and 4 weeks of consolidation, respectively (Figure 6D), suggesting the bone-regenerating effect of AS-IV on the rat DO model.

### Vascularized bone regeneration enhanced by AS-IV in the distraction area

**Figure 7:**
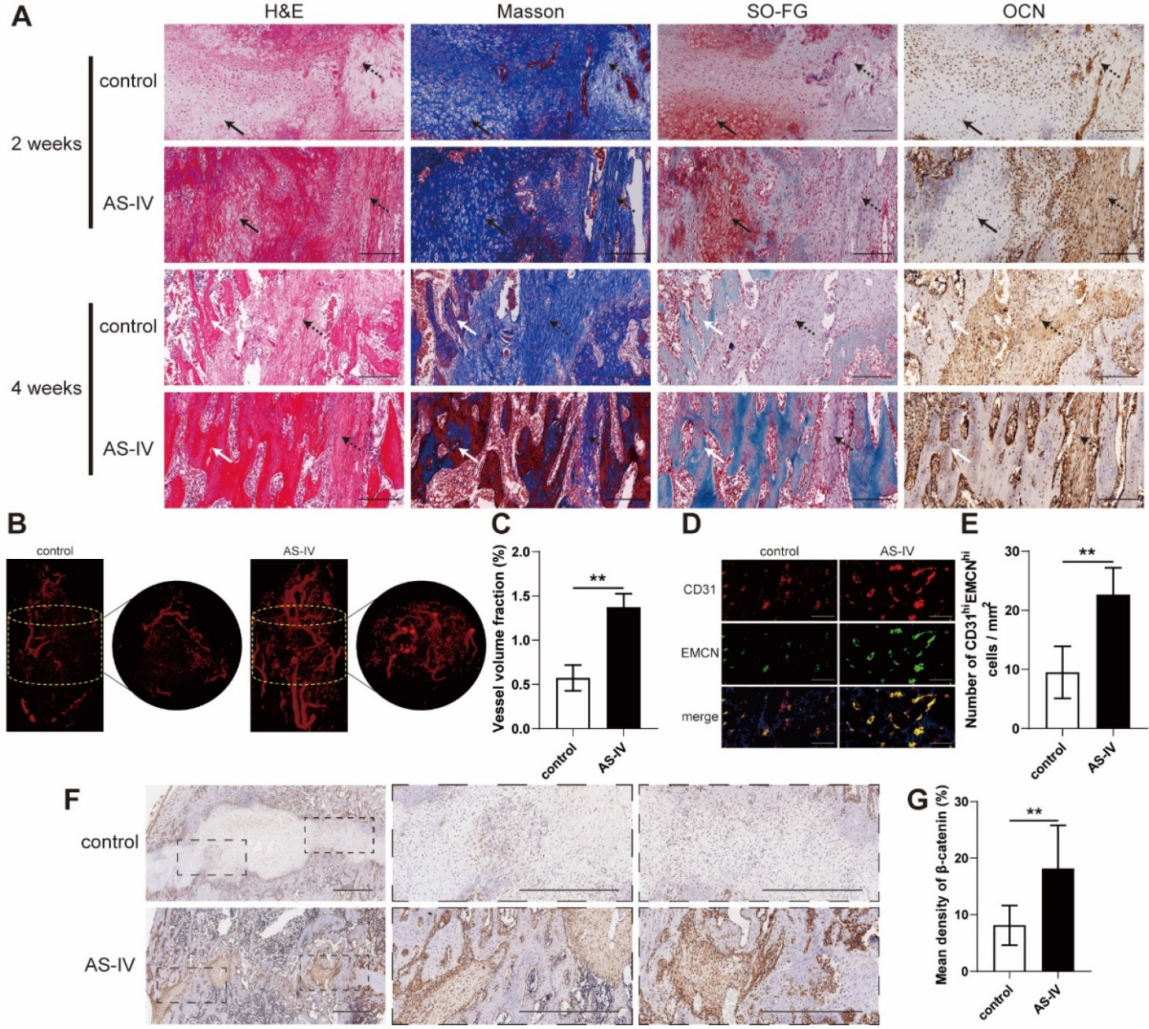
** AS-IV improves vascularized bone regeneration in the tibial distraction zone. (A)** Representative images of H&E, Masson, and Safranin O-Fast Green staining and immunohistochemical analysis of OCN. Scale bar: 200 µm. Black arrows, cartilaginous tissue. Dotted arrows, fibrous-like tissue. White arrows, newly formed trabecular bone.** (B)** Micro-CT observation of the newly formed blood vessels perfused with Microfil in the distraction regions. **(C)** Quantitative analysis of the vessel volume fractions within the distraction gaps from the two groups. **(D)** Immunofluorescence staining images of CD31 and EMCN for the distraction area sections from the two groups. Scale bar: 100 µm. **(E)** Quantitative analysis of CD31^hi^EMCN^hi^ cells per mm^2^ from the staining results. **(F)** Immunohistochemical analysis of β-catenin in control and ADM2 groups. Scale bar: 500 µm.** (G)** Quantitative analysis of the immunohistochemical staining of β-catenin. The data were confirmed by Student's *t*-test between control group and AS-IV group and are presented as the means ± SD.^ **^P < 0.01.

The H&E, Masson's trichrome, and SO-FG staining of the distraction regenerates revealed various amounts of newly formed trabecular bone, cartilaginous tissue, and fibrous-like tissue, parallel with the distraction forces (Figure 7A). However, distraction regenerates treated with AS-IV exhibited enhanced bone regeneration during consolidation phase in comparison to the control group, which was evident through the presence of more mature trabecular bone, less fibrous-like tissue, and more intense immunohistochemical staining of OCN in the AS-IV group (Figure 7A).

Regarding neovascularization, there were more neo-vessels, as confirmed by elevated vessel volume fraction, growing into the distraction area of the AS-IV group than in the control group (Figure 7B, C). Additionally, CD31 and EMCN immunofluorescence double staining revealed a greater amount of CD31^hi^Emcn^hi^ cells in the distraction regenerates of the AS-IV group (Figure 7D, E). These data suggest that enhancing the coupling of osteogenesis and angiogenesis might be another important mechanism by which AS-IV accelerates bone regeneration in DO, besides its direct stimulation of osteogenesis. Additionally, immunohistochemical analysis revealed higher expression of β-catenin within the distraction regenerates of the AS-IV group (Figure 7F, G), suggesting that β-catenin signaling contributed to the enhancement in the coupling of osteogenesis and angiogenesis induced by AS-IV treatment during DO.

## Discussion

In the present study, we found that AS-IV improved the angiogenic ability of CM from RANKL-induced macrophages by inhibiting the formation of multinucleated osteoclasts and preserving preosteoclasts. Furthermore, we demonstrated that the angiogenic effect of CM^RANKL+AS-IV^ was achieved, at least in part, by the production of PDGF-BB from the preosteoclasts. Moreover, we verified the improvement effects of AS-IV on the viability, osteogenic differentiation, and angiogenic gene expression of BMSCs. Interestingly, both biological effects of AS-IV on osteoclastogenesis and osteogenesis were attributed mostly to the activation of the AKT/GSK-3β/β-catenin pathway induced by AS-IV. *In vivo,* the improvement effects of AS-IV on bone regeneration and vessel expansion in DO rats were also verified. To the best of our knowledge, this is the first study to substantiate that AS-IV can enhance the coupling of osteogenesis and angiogenesis by activating the AKT/GSK-3β/β-catenin pathway.

**Figure 8:**
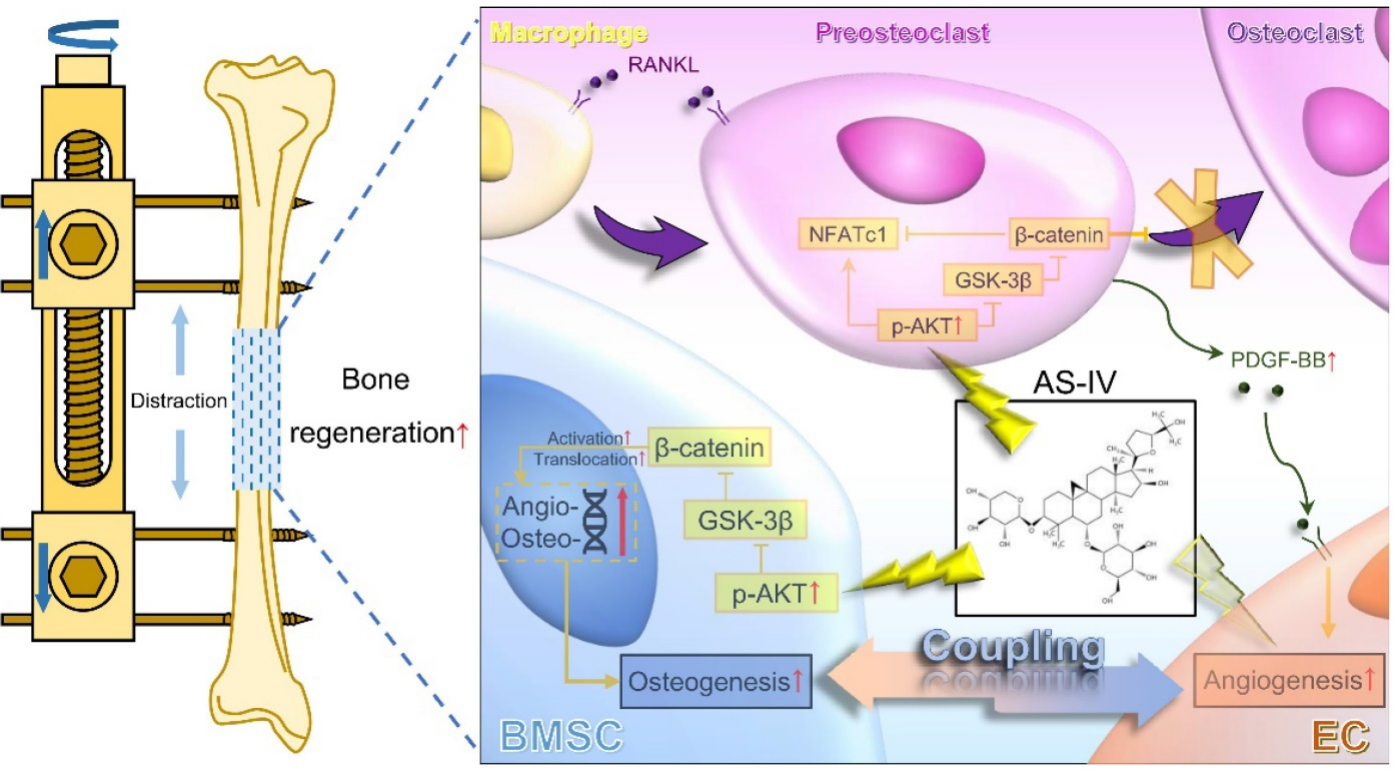
** Working model of AS-IV promoting osteogenesis along with preosteoclast-induced angiogenesis during DO by activating AKT/GSK-3β/β-catenin pathway.** Under the administration of AS-IV, AKT/GSK-3β/β-catenin signaling is activated in BMSCs and macrophages. In BMSCs, the osteogenic potential is enhanced, and the expression of angiogenic genes is up-regulated. Additionally, in RANKL-induced macrophages, the activated AKT/GSK-3β/β-catenin signaling inhibits the maturation of multinucleated osteoclasts, increasing the production of PDGF-BB from preosteoclasts, thus enhancing the angiogenesis of ECs. In conclusion, AS-IV could directly promote osteogenesis and indirectly improve angiogenesis by activating AKT/GSK-3β/β-catenin signaling in BMSCs and macrophages simultaneously, thus enhancing the coupling of osteogenesis and angiogenesis and accelerating bone regeneration during DO. AS-IV: astragaloside IV. AKT: protein kinase B. GSK-3β: glycogen synthase kinase-3β. BMSCs: bone marrow derived mesenchymal stem cells. DO: distraction osteogenesis. RANKL: receptor activator for nuclear factor κB ligand. PDGF-BB: platelet-derived growth factor-BB. ECs: endothelial cells.

Considerable evidence has shown that vascular endothelial cells play a vital role in the regulation of osteogenesis in the skeletal system 10. As knowledge about the coupling of osteogenesis and angiogenesis expansion, preosteoclasts and osteoblasts have been identified as important regulatory cells to enhance the expansion of type-H vessels 15, 33, 34. In other words, in addition to directly regulating endothelial cells, inhibition of osteoclastogenesis and promotion of osteogenesis could be employed as indirect therapeutic strategies to promote angiogenesis, which could accelerate bone regeneration in DO. Previous studies have verified that AKT activation could stimulate the nuclear translocation of β-catenin through phosphorylation and inactivation of GSK-3β 32, 35, 36. Of note, AS-IV has been widely reported to activate AKT signaling 24, 37, 38; therefore, we hypothesized that AS-IV would activates the Wnt pathway, which inhibits osteoclastogenesis and promotes osteogenesis in macrophages and BMSCs. As the direct angiogenic effect of AS-IV has been widely reported, the present study focused on investigating the effects of AS-IV-mediated activation of the AKT/GSK-3β/β-catenin pathway on osteoclastogenesis and osteogenesis. Thus, for the first time, AS-IV was shown to act as an active molecule that significantly promotes the coupling of osteogenesis and angiogenesis (Figure 8).

An investigation has shown that AS-IV could prevent titanium-induced osteolysis by inhibiting multinucleated osteoclast formation 27. However, in the process of bone regeneration, prolonged consolidation is normally irrelevant to osteoclast overactivation, and the dynamic balance of bone resorption and formation is indispensable 39-41. Hence, excessive inhibition of osteoclast activity is not routinely considered to be an appropriate strategy to promote bone regeneration. However, excessive inhibition of osteoclastogenesis may exert a negative effect on the healing process 42-44. Nonetheless, inhibiting the formation of multinucleated osteoclasts to increase the production of preosteoclast PDGF-BB and thus indirectly promoting the coupling of osteogenesis and angiogenesis has been shown to have a promising therapeutic potential 33, 45. Huang et al. utilized harmine to suppress osteoclast formation, eventually enhancing the expansion of type-H vessels and the osteogenic response in an ovariectomy-induced osteoporotic mouse model, even though harmine exhibited a direct inhibitory effect on angiogenesis of endothelial cells *in vitro* 19. In the present study, we verified that an appropriate concentration of AS-IV possesses specific biological functions in inhibiting osteoclastogenesis and promoting preosteoclast PDGF-BB-induced angiogenesis by activating AKT/GSK-3β/β-catenin signaling. We believe that these biological functions of AS-IV are mostly attributed to the increased population of preosteoclasts, which are the main physiological source of PDGF-BB within the skeleton system 33. However, the expression of PDGF-B was reported to be directly regulated by β-catenin in endothelial cells 46, indicating that the intrinsically elevated expression of *PDGF-BB* on transcription level induced by the activation of β-catenin in preosteoclasts could be another alternative explanation for these biological functions of AS-IV, although this hypothesis remains to be verified by further studies on the interaction between β-catenin and the promoter of *PDGF-B* gene in preosteoclasts. Nevertheless, combined with its direct angiogenic effects reported in the previous studies, AS-IV could be an ideal natural molecule for assisting bone regeneration in DO by directly and indirectly promoting angiogenesis.

Compared to angiogenesis, the osteogenic differentiation of MSCs plays a more direct and central role in bone regeneration 47-49. A previous study demonstrated that AS-IV combined with centrifugation pressure could promote the osteogenic differentiation of OCT-1 cells 28. However, the effect and mechanism of AS-IV on the osteogenic potential of BMSCs remain uninvestigated, along with its *in vivo* effects on bone regeneration. In the present study, we verified that AS-IV could promote the viability and osteogenic capacity of BMSCs. Moreover, the AKT/GSK-3β/β-catenin pathway was activated by AS-IV treatment in BMSCs, while the promoting effects of AS-IV *in vitro* could be at least partially attenuated by an AKT antagonist, as expected, indicating that AS-IV facilitated the viability and osteogenic differentiation of BMSCs via the AKT/GSK-3β/β-catenin pathway. In addition, the AS-IV-induced activation of the AKT/GSK-3β/β-catenin pathway also elevated the angiogenic gene expression of BMSCs during osteogenic induction. Notably, mature osteoblasts are inherently more capable of promoting angiogenesis than their precursor cells, according to the theory of the coupling of osteogenesis and angiogenesis 15, 50, 51. Thus, it seems dubious whether this effect of AS-IV is attributed to the increased population of osteoblasts or the improved pro-angiogenic activity of BMSCs and/or osteoblasts. As revealed by our results, the gene expression of *VEGFA* was solely downregulated during osteogenic differentiation. However, the AS-IV treatment not only promoted osteogenic differentiation of BMSCs, which was supposed to further downregulate the gene expression of *VEGFA*, but also rescued *VEGFA* expression via the AKT/Wnt pathway, suggesting that the pro-angiogenic activity of osteoblast lineage cells was indeed stimulated by AS-IV treatment.

To date, very few side effects of AS-IV have been reported, except for mild maternal toxicity and fetotoxicity 52, 53. However, according to our* in vitro* results, a high concentration (80 μM) of AS-IV failed to further promote osteogenic differentiation, but relatively inhibited the proliferation of BMSCs. In addition, a higher dose of AS-IV excessively inhibited osteoclastogenesis, thereby failing to play an optimal role in promoting the production of PDGF-BB, suggesting that it may instead affect the bone remodeling process. Moreover, a previous study demonstrated that as the concentration of AS-IV increased, the proliferation rate of endothelial cells first increased and then decreased under hypoxic conditions 23. Based on the existing literature, although AS-IV administration is relatively safe, further research should be devoted to investigating the metabolic characteristics and toxicity for the clinical application of AS-IV.

The present study has several limitations. First, the detailed mechanisms underlying the ability of AS-IV to activate AKT remain to be fully elucidated. Second, detailed pharmacokinetic characteristics and pharmacological safety parameters, including the serum levels of liver and kidney function indicators, which are critical for clinical translational studies have not been well explored. Third, the activation level of the AKT/Wnt pathway was not examined *in vivo,* and pathway inhibitor intervention was not applied to further verify the mechanism by which AS-IV promotes bone regeneration in DO. Future work should target the molecular mechanisms underlying the promotive effect of AS-IV and explore its optimal administration in large animal models.

## Conclusions

This study demonstrates that AS-IV improves the preosteoclast PDGF-BB-induced angiogenesis along with the viability, osteogenic differentiation, and pro-angiogenic potential of BMSCs via the AKT/GSK-3β/β-catenin pathway, revealing AS-IV as a novel molecule to accelerate bone regeneration during DO. Moreover, our study also provides a novel therapeutic strategy for accelerating bone regeneration in DO, which is enhancing the coupling of osteogenesis and angiogenesis by activating AKT/GSK-3β/β-catenin pathway.

## Figures and Tables

**Table 1:** Primer sequences

Gene	Forward (5'-3')	Reverse (5'-3')
*ALP*	CCGCAGGATGTGAACTACT	GGTACTGACGGAAGAAGGG
*Ang-2*	GAAGAAGGAGATGGTGGAGAT	CGTCTGGTTGAGCAAACTG
*Ang-4*	GCTCCTCAGGGCACCAAGTTC	CACAGGCGTCAAACCACCAC
*Atp6v0d2*	GGAAGCCCAGTAAACAGAAC	CCAGTGAGCAGGAAGTCATA
*CTSK*	CCAGAATCTTGTGGACTGTGT	CATCTTCAGAGTCAATGCCTC
*GAPDH (mouse)*	AAATGGTGAAGGTCGGTGTG	AGGTCAATGAAGGGGTCGTT
*GAPDH (Rat)*	ATGGCTACAGCAACAGGGT	TTATGGGGTCTGGGATGG
*OCN*	CAGACAAGTCCCACACAGCA	CCAGCAGAGTGAGCAGAGAGA
*OPN*	GGCCGAGGTGATAGCTT	CTCTTCATGCGGGAGGT
*OSX*	GGAAAAGGAGGCACAAAGAA	CAGGGGAGAGGAGTCCATT
*PDGF-BB*	CAGGGTGAGCAAGGTTGTAAT	TTTAGGGTGTGAACAGCAGTC
*Runx2*	ACTTCCTGTGCTCGGTGCT	GACGGTTATGGTCAAGGTGAA
*SDF-1*	GCGTCTATGTCTTGTTTGGAA	TACCTCATACACAGCCTTTGC
*Slit3*	GCTAAACCAGACCCTGAACCT	ACTGTTGATGCCCACTGCT
*VEGFA*	CACGACAGAAGGGGAGCAGAAAG	GGCACACAGGACGGCTTGAAG

## Abbreviations

AKT: protein kinase B
ALP: alkaline phosphatase
Ang-2: angiopoietin-2
Ang-4: angiopoietin-4
ARS: alizarin red S
AS-IV: astragaloside IV
BMD: bone mineral density
BMSC: bone marrow mesenchymal stem cell
BSA: bovine serum albumin
BV/TV: bone volume/tissue volume
CD31: platelet endothelial cell adhesion molecule-1
CM: conditioned media
CT: computed tomography
CTSK: cathepsin K
DAPI: 4'6-diamidino-2-phenylindole
DMSO: dimethyl sulfoxide
DO: distraction osteogenesis
EDTA: ethylene diamine tetraacetic acid
ELISA: enzyme-linked immunosorbent assay
EMCN: endomucin
E-modulus: modulus of elasticity
FBS: fetal bovine serum
GSK-3β: glycogen synthase kinase-3β
H&E: hematoxylin-eosin
HRP: horseradish peroxidase
NFATc1: nuclear factor of activated T-cells
OCN: osteocalcin
OIM: osteogenic induction media
OPN: osteopontin
OSX: osterix
P/S: penicillin-streptomycin
PBS: phosphate-buffered saline
PDGF-BB: platelet-derived growth factor-BB
PFA: paraformaldehyde
qRT-PCR: quantitative real-time polymerase chain reaction
RANKL: nuclear factor κB ligand
Runx2: runt-related transcription factor 2
SD: Sprague-Dawley
SDF-1: stromal cell derived factor-1
Slit3: slit homolog 3
SO-FG: safranine O-fast green
TRAP: tartrate-resistant acid phosphatase
VEGF-A: vascular endothelial growth factor-A
Wnt: wingless/integrated
